# Is waist-to-height ratio the best predictive indicator of cardiovascular disease incidence in hypertensive adults? A cohort study

**DOI:** 10.1186/s12872-022-02646-1

**Published:** 2022-05-11

**Authors:** Shu Zhang, Xin Fu, Zhi Du, Xiaofan Guo, Zhao Li, Guozhe Sun, Ying Zhou, Hongmei Yang, Shasha Yu, Liqiang Zheng, Yingxian Sun, Xingang Zhang

**Affiliations:** 1 Department of Cardiovascular Medicine, The First Hospital of China Medical University, Shenyang, 110001 Liaoning China; 2 Department of Orthopedics, The Second Affiliated Hospital of Shenyang Medical College, Shenyang, China; 3 Clinical Epidemiology, Library Shengjing Hospital of China Medical University, Shenyang, China

**Keywords:** Cardiovascular disease, Hypertensive adults, WHtR

## Abstract

**Background:**

Cardiovascular disease (CVD) brings high mortality and economic burden to patients, especially in rural areas. Simple, low-cost abdominal adiposity measures may help identify individuals with increased CVD risk. It is unclear that which obesity indices is the best to predict CVD in hypertensive people.

**Methods:**

Northeast China Rural Cardiovascular Health Study (NCRCHS) is a prospective cohort study in a general population in Northeast China. The study examined the cardiovascular health from 2013 to 2015, and follow-up captured the CVD incidence in 2018. Baseline waist-to-height ratio (WHtR), waist circumference (WC), waist-to-hip (WHR)and body mass index (BMI) were calculated and analyzed in relation to the CVD incidence.

**Results:**

A total of 4244 hypertensive adults without pre-existing CVD at baseline were included in this analysis (age 35–92 years; 2108 men). Over a median follow-up of 4.66 years, a total of 290 CVD cases (6.83%) were documented during the follow-up. Baseline WHtR showed a significant positive association with CVD incidence, even after adjusting for age, sex, diabetes, drinking, smoking, SBP, DBP, Triglyceride, HDL-C, LDL-C, and TC (Hazard Ratios per SD of WHtR ranging from 1.03 to 1.31, *p* = 0.017). Reclassification and discrimination analyses indicated WHtR addition could improve the conventional model for predicting adverse outcomes within 4 years. Moreover, WHtR predicted the CVD incidence better than other obesity indices (BMI, WC, WHR).

**Conclusion:**

These findings support a positive association between WHtR and CVD incidence in CVD-free hypertensive adults. WHtR can be used to predict CVD incidence in hypertensive adults.

**Supplementary Information:**

The online version contains supplementary material available at 10.1186/s12872-022-02646-1.

## Background

Cardiovascular disease (CVD), has becoming a public health challenge strongly linked with the aging global population [1–3]. In China, CVD is the leading cause of death. It accounts for 45.01% of total deaths in rural areas in 2015, while the proportion of urban areas is relatively lower than rural areas. The number of CVD patients is on the rise and predicted to increase substantially over the next 10 years [4].

Obesity, particularly abdominal, is a key risk factor of cardiovascular disease (CVD) [5–7]. Using anthropometric indices to categorize obesity such as waist circumference (WC), the waist-to-height ratio (WHtR), body mass index (BMI), and the waist-to hip ratio (WHR) [8] are the simplest and the most cost-effective methods recommended in clinical practice and epidemiological research, especially in developing countries. Some studies demonstrated that WHtR was better for predicting CVD risk, but it is unclear in the hypertensive adults [9, 10].

Hypertension (HTN) is regarded as a serious public health problem [11–13]. The prevalence of HTN has been increasing all over the world [14–16]. However, it is not known that which anthropometric indices are the best to predict CVD in people with hypertension. Thus, elucidation of the best predictive indicator of cardiovascular disease incidence in hypertension adults is of great significance.

In the present study, we aimed to determine the role of WHtR as a predictor of CVD incidence in the NCRCHS (the Northeast China Rural Cardiovascular Health Study) cohort of hypertensive adults without previous CVD, and compare its discriminating ability against other commonly anthropometric indices of central obesity (i.e., WC, WHR and BMI).

## Methods

### Study population

Northeast China Rural Cardiovascular Health Study (NCRCHS) is a cohort study in a general population. The methods of the study, including design, personnel recruitment, and laboratory techniques, have been described in previous publications [16, 17]. Between January 2013 and August 2013, 11,956 subjects aged ≥ 35 years were recruited from rural areas of Liaoning province. Subsequently, subjects were invited to attend follow-up visits in 2015 and 2018, and 6017 hypertensive participants were consented and eligible for the follow-up study. A total of 5249 participants of hypertension completed at least one follow-up visit. In the present study, we excluded baseline history of coronary heart disease (n = 355) and stroke (n = 590), and missing data (n = 60). Eventually, data from 4244 participants were available for analysis. The Ethics Committee of the First Hospital of China Medical University (Shenyang, China) approved the study. All participants wrote the informed consent.

### Data collection

Data was collected during a single clinic visit by cardiologists and trained nurses using a standard questionnaire by face-to-face interview. During data collection, our inspectors had further instructions and support.

All participants were asked about the current status of smoking, drinking and the history of diseases. All participants were performed according to the BMI levels of China (BMI < 18.5 kg/m^2^, 18.5 kg/m^2^ ≤ BMI < 24 kg/m^2^, 24 kg/m^2^ ≤ BMI < 30 kg/m^2^, BMI ≥ 30 kg/m^2^). WC divided by height is the waist-to-height ratio. We categorized WHtR according to the Ashwell's reports. The reference group was the participants with WHtR between 0.40 and 0.50 [18–20].

According to American Heart Association protocol, blood pressure was measured three times at 2-min intervals after at least 5 min of rest using a standardized automatic electronic sphygmomanometer (HEM-907; Omron). The mean of three blood pressure measures was calculated and used in all analyses. Hypertension was defined as a mean DBP ≥ 90 mmHg, and/or a mean SBP ≥ 140 mmHg, and/or use of the antihypertensive medication in the previous 2 weeks [21, 22]. Diabetes mellitus was defined as FBG ≥ 7.0 mmol/l and/or self-reported physician-confirmed diagnosis [23]. Fasting blood samples were collected after at least 10 h of fasting. Blood samples were taken from an antecubital vein into BD Vacutainer tubes containing ethylenediaminetetraacetic acid. Serum was subsequently isolated from the whole blood, and all serum samples were frozen at − 80 °C for testing at a central, certified laboratory. We used the Olympus AU640 auto-analyzer (Olympus, Kobe, Japan) for analyzing blood biochemical indexes. All blinded duplicate samples were used for these analyses.

### Judgment and definition of clinical outcomes

We collected all available clinical information about possible diagnoses or mortality, including data from medical records and death certificates. CVD was defined as stroke or Coronary heart disease (CHD). Stroke were diagnosed by neurologists following the examination of computed tomography and magnetic resonance imaging data in accordance with World Health Organization (WHO) criteria [24]. CHD was defined as a diagnosis of angina requiring hospitalization, myocardial infarction (MI), revascularization procedure and CHD-related mortality [25].

### Statistical analysis

Continuous variables were presented as means and SDs and categorical variables were reported as frequencies and percentages in each group. Differences between categories were evaluated using the t test, or the Chi-Square test. Kaplan–Meier method was used to calculate the cumulative incidence for adverse events, and log-rank test was used to compare differences. We used Cox proportional hazards models to estimate the Hazard ratios (HR) and 95% confidence intervals (95% CI) for the association between anthropometric obesity indicators and CVD event. To evaluate the improvement in risk prediction for adverse outcomes by adding WHtR to the conventional model (including age, sex, current smoking, current drinking, SBP, DBP, TC, HDL-C, LDL-C, triglyceride, and diabetes), net reclassification improvement (NRI) and integrated discrimination improvement (IDI) was calculated for CVD prediction models respectively (conventional model vs. conventional model + WHtR). The calculation method is IDI = (Pnew, events-Pold, events)-(Pnew, non-events-Pold, non-events). With the larger value of IDI, the new model has the better prediction ability.

SPSS software version 22.0 was used for statistical analysis and statistical software packages R (http://www.R-project.org, The R Foundation). *P* < 0.05 was considered to be statistically significant.

## Results

### Baseline characteristics of the study sample according to CVD incidence

In this study, there are 6017 hypertensive participants consented and eligible for the follow-up study. A total of 5249 participants of hypertension completed at least one follow-up visit. In the current study, we excluded baseline history of coronary heart disease (n = 355) and stroke (n = 590), and missing physical indicators (n = 60). Table 1 presents the baseline characteristics of participants according to the CVD incidence. CVD was defined as stroke or Coronary heart disease (CHD). 4244 participants (2108 men and 2136 women, mean age 56.26 ± 10.15 years) were included in this cohort study (Additional file 1: Figure S1). During a median follow-up of 4.66 years, 290 participants (6.83%) incident stroke or CHD (crude incidence rate, 14.66 incident stroke or CHD per 1000 person-years).

**Table 1:** Baseline characteristics of the study sample

Variable	Without CVD (N = 3954)	With CVD (N = 290)	*P* value
Age (years)	55.85 (± 10.10)	61.85 (± 9.26)	< 0.001
Male [n (%)]	1945 (50.8%)	163 (56.2%)	0.021
Current smoking [n (%)]	1406 (35.6%)	118 (40.8%)	0.090
Current drinking [n (%)]	1065 (26.9%)	87 (30.0%)	0.287
Body Mass Index (kg/m^2^)	25.55 (± 3.58)	25.79 (± 3.54)	0.268
Waist circumference (cm)	84.39 (± 9.52)	85.80 (± 10.49)	0.016
Waist-to-hip ratio	0.87 (± 0.07)	0.89 (± 0.10)	< 0.001
Waist-to-height ratio	0.53 (± 0.06)	0.54 (± 0.06)	0.001
SBP (mmHg)	157.90 (± 18.18)	168. 47 (± 23.21)	< 0.001
DBP (mmHg)	88.69 (± 10.78)	91.47 (± 14.12)	0.001
TC (mmol/L)	5.40 (± 1.09)	5.55 (± 1.11)	0.023
LDL-C (mmol/L)	3.08 (± 0.85)	3.22 (± 0.91)	0.017
HDL-C (mmol/L)	1.45 (± 0.41)	1.45 (± 0.45)	0.909
Triglyceride (mmol/L)	1.76 (± 1.65)	1.77 (± 1.53)	0.978
Diabetes [n (%)]	192 (4.9%)	19 (6.6%)	0.253

Date are presented as mean ± standard deviation, or n (%), as appropriate

SBP, systolic blood pressure; DBP, diastolic blood pressure; TC, total cholesterol; LDL-C, low-density lipoprotein cholesterol; HDL-C, high-density lipoprotein cholesterol; CVD, cardiovascular disease

The group of participants who developed CVD during the study follow-up consisted mainly of older men and exhibited higher anthropometric indices/ratios of total and central obesity (BMI, WC, WHR, WHtR), compared to those who remained CVD-free. Furthermore, this group had higher baseline DBP, SBP levels and lipids (TC and LDL-C) (all *p* < 0.05; Table 1). The mean WHtR value at baseline was 2% higher in the group of participants who developed a CVD event than in those who remained CVD-free (*p* < 0.05, Table 1).

### Baseline WHtR in relationship to the CVD incidence

**Fig. 1:**
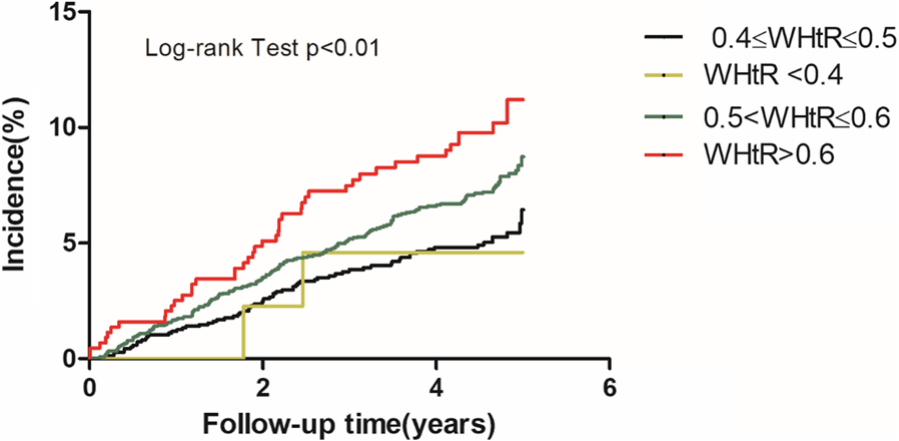
Unadjusted Kaplan–Meier curves for incident adverse events stratified by waist-to-height ratio. WHtR, waist-to-height ratio

The four groups of the Kaplan–Meier survival curves were showed in Fig. 1. The figure showed that the cumulative CVD incidence in the group with WHtR > 0.60 was highest and was much higher than that in the group with 0.4 ≤ WHtR < 0.5. (p for Log-rank test = 0.003, *p* < 0.05) Sex didn’t have statistically significant interaction in the association between obesity indicators and CVD incidence.

Table 2 shows the HRs for CVD according to the value of four abdominal adiposity indices. Univariate HR of CVD for people with WHtR > 0.60 by 1.98-fold (*P* < 0.001), compared with the group with 0.4 ≤ WHtR < 0.5. After adjustment for sex, age, current drinking, current smoking, TC, HDL-C, LDL-C, triglyceride, diabetes, SBP and DBP, the HR (95% CI) for participants with WHtR 0.60 increased was 1.87 (95% CI 1.23–2.83) for CVD (*P* = 0.003). Moreover, the participants with 0.50 ≤ WHtR < 0.60 still had a significant difference comparing with the reference group. The multivariate-adjusted HR (95% CI) for 0.50 ≤ WHtR < 0.60 was 1.45 (95% CI: 1.09–1.94) for CVD. However, the other three abdominal adiposity indices didn’t show the such significant results.

**Table 2:** Associations between risks of adverse outcomes and different values of waist-to-height ratio, Body Mass Index, waist circumference and waist-to-hip ratio

All participants	Unadjusted HR (95%CI)	*P* value	Adjusted HR (95%CI)	*P* value
WHtR				
WHtR < 0.4	0.83 (0.20–3.37)	0.790	0.98 (0.24–3.99)	0.973
0.4 ≤ WHtR < 0.5	1	–	1	–
0.5 ≤ WHtR < 0.6	1.42 (1.08–1.87)	0.013	1.45 (1.09–1.94)	0.012
WHtR > 0.6	1.98 (1.35–2.89)	< 0.001	1.87 (1.23–2.83)	0.003
BMI				
BMI < 18.5	1.42 (0.33–2.40)	0.496	1.03 (0.38–2.82)	0.954
18.5 ≤ BMI < 24	1	–	1	–
24 ≤ BMI < 30	1.07 (0.83–1.38)	0.601	1.12 (0.86–1.47)	0.399
BMI > 30	1.42 (0.96–2.10)	0.083	1.52 (0.99–2.33)	0.055
WC				
1st quartile	1	–	1	–
2nd quartile	1.38 (0.98–1.95)	0.063	1.36 (0.96–1.92)	0.081
3rd quartile	1.34 (0.96–1.88)	0.086	1.28 (0.90–1.81)	0.170
4th quartile	1.45 (1.03–2.05)	0.033	1.33 (0.92–1.92)	0.133
WHR				
1st quartile	1	–	1	–
2nd quartile	1.58 (1.12–2.23)	0.009	1.47 (1.04–2.08)	0.031
3rd quartile	1.62 (1.13–2.32)	0.009	1.43 (0.99–2.07)	0.059
4th quartile	1.85 (1.31–2.62)	< 0.001	1.41 (0.98–2.03)	0.064

Adjusted for age, sex, current smoking, current drinking, TC, HDL-C, LDL-C,triglyceride, diabetes, SBP and DBP

WHtR, waist-to-height ratio; BMI, Body Mass Index; WHR, waist-to-hip ratio; WC, waist circumference; SBP, systolic blood pressure; DBP, diastolic blood pressure; TC, total cholesterol; LDL-C, low-density lipoprotein cholesterol; HDL-C, high-density lipoprotein cholesterol; CVD, cardiovascular disease

### Reclassification and discrimination statistics for adverse outcomes within 4 years by waist-to-height ratio, Body Mass Index, waist circumference and waist-to-hip ratio

Furthermore, we evaluated whether adding WHtR to the conventional model could improve prediction performance. Fortunately, the IDI value and NRI value suggested that the model after addition of WHtR led to a significant improvement in predicting incident stroke or CHD within 4 years (Table 3).

**Table 3:** Reclassification and discrimination statistics for adverse outcomes within 4 years by waist-to-height ratio, Body Mass Index, waist circumference and waist-to-hip ratio

	NRI(95% CI)	IDI	*P*
WHtR	0.05 (− 0.01 to 0.12)	0.0026	0.01
BMI	− 0.01 (− 0.05 to 0.03)	0.0009	0.09
WC	− 0.01 (− 0.05 to 0.02)	0.0007	0.14
WHR	− 0.01 (− 0.05 to 0.04)	0.0013	0.04

Conventional model: age, sex, current smoking, current drinking, TC, HDL-C, LDL-C, triglyceride, diabetes, SBP and DBP

WHtR, waist-to-height ratio; BMI, Body Mass Index; WHR, waist-to-hip ratio; WC, waist circumference; SBP, systolic blood pressure; DBP, diastolic blood pressure; TC, total cholesterol; LDL-C, low-density lipoprotein cholesterol; HDL-C, high-density lipoprotein cholesterol

The result showed that the NRI value of WHtR was 0.05(more than 0 indicating improvement). The IDI value of WHtR was 0.0026 (*p* = 0.01). Based on these models, WHtR exhibited better predictive value for the CVD incidence as revealed through the IDI value and NRI value (the higher the better), than other common anthropometric indices. Similarly, baseline WHtR was also a better predictor of the CVD than BMI, WHR and WC.

## Discussion

WHtR was associated with risk of the CVD in people with hypertension in the prospective cohort study. Notably, this positive association remained significant even after adjusting for various CVD risk factors. Moreover, in the performed comparisons of the predictive value of WHtR on the CVD incidence, WHtR was better than other common anthropometric indices of obesity (BMI, WHR and WC). WHtR exhibited better predictive value for the CVD incidence than the others.

CVD is a public health challenge. The number and mortality of CVD patients is on the rise. The incidence rate of CVD in rural areas was higher than the Chinese average level. Therefore, the results of our study will have important clinical predictive significance. Especially the rural population, their income is generally low, WHtR without cost is the simplest and effective method in clinical practice.

WHtR is a rapid application tool that can help predict CVD risk, and then reduce the CVD incidence. Our results also indicated that addition of WHtR could improve the conventional model for predicting adverse outcomes within 4 years. In addition, our results appear to be stable because the value of WHtR for predicting adverse events remained constant. It is the best indicator of obesity that predicts CVD risk in the population with hypertension.

Recently, there are some studies that reveal the relationship between WHtR and CVD [8, 26, 27]. The study of Gelber pointed that WHtR had the highest association with CVD in male [28]. In addition, the superiority of WHtR in detecting the risk of CVD in children and adolescents has been reported [29]. Other studies demonstrated that WHtR was better for predicting CVD risk [30, 31]. However, the result is from mostly cross-sectional studies, and the study population is not hypertensive.

Our Population in young adults where > P90 over three measures. The strength of current study is that it is a large prospective cohort study. It is the first study to show the association between CVD and WHtR in hypertensive people. Moreover, confounding factors were adequately adjusted and the results were still stable. Nevertheless, our study also has limitations. Cohort sampling loss should be considered a limitation of the study. Our study is a Chinese cohort, which limits the generalizability of our findings to other ethnic groups.

## Conclusion

The present study emerges evidence suggesting that WHtR may constitute a simple and accurate prognostic marker of CVD risk as compared to other obesity-related indices in hypertensive people. Indeed, the present findings offer new prospective data suggesting that WHtR exhibits a positive association with the CVD incidence in Asiatic adults from the hypertensive population without pre-existing CVD. WHtR appears to be a much better predictor of CVD risk than the other anthropometric indices of total and central obesity. Future studies are still required to further evaluate the association between WHtR and CVD in different ethnic and patient populations.

## Supplementary Information

**Additional file 1. Supplemental figure S1.** Study flow chart.

## Data Availability

Data are available upon reasonable request. Our data will not be shared because some articles are still being submitted. Corresponding author will be contact for the data.

## Abbreviations

WHtR: Waist-to-height ratio
BMI: Body Mass Index
WHR: Waist-to-hip
WC: Waist circumference
CVD: Cardiovascular disease
NCRCHS: Northeast China Rural Cardiovascular Health Study
TC: Total cholesterol
CHD: Coronary heart disease
NRI: Net reclassification improvement
IDI: Integrated discrimination improvement
