# Supplementary figures and images for: Is waist-to-height ratio the best predictive indicator of cardiovascular disease incidence in hypertensive adults? A cohort study
Source: BMC Cardiovasc Disord. 2022 May 11;22:214. doi: 10.1186/s12872-022-02646-1 (PMC9092683; doi:10.1186/s12872-022-02646-1)

Supplemental figure S1. Study flow chart.


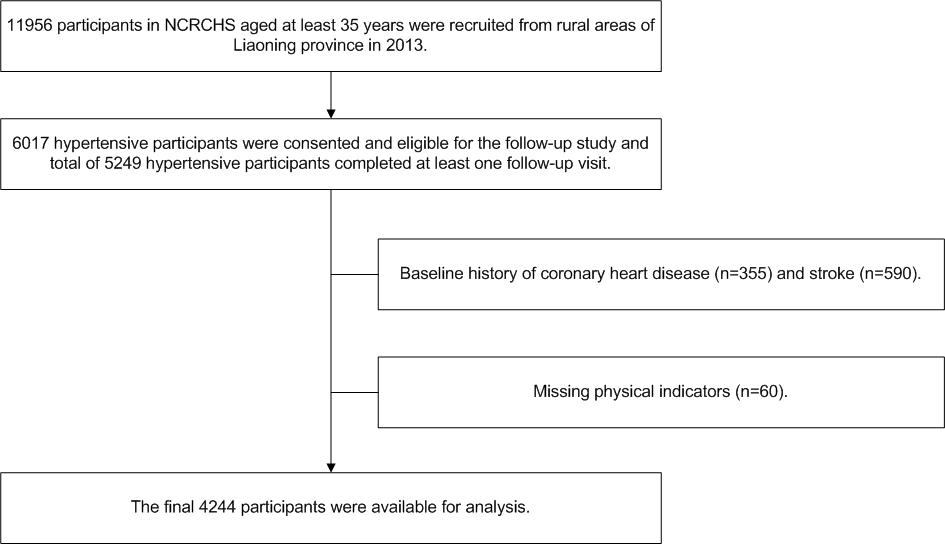

Supplement: Supplementary file 1 — Additional file 1. Supplemental figure S1. Study flow chart. [file 12872_2022_2646_MOESM1_ESM.docx]
